# Can We Motivate Students to Practice Physical Activities and Sports Through Models-Based Practice? A Systematic Review and Meta-Analysis of Psychosocial Factors Related to Physical Education

**DOI:** 10.3389/fpsyg.2019.02115

**Published:** 2019-10-10

**Authors:** Manuel Jacob Sierra-Díaz, Sixto González-Víllora, Juan Carlos Pastor-Vicedo, Guillermo Felipe López-Sánchez

**Affiliations:** ^1^EDAF Group, Didactics of Musical, Plastic and Physical Education Department, Faculty of Education of Cuenca, University of Castilla-La Mancha, Cuenca, Spain; ^2^EDAF Group, Didactics of Musical, Plastic and Physical Education Department, Faculty of Education of Albacete, University of Castilla-La Mancha, Albacete, Spain; ^3^Faculty of Sport Sciences, University of Murcia, Murcia, Spain

**Keywords:** self-determination theory, self-determined motivation, flow state, basic psychological needs (BPNs), physical education engagement, child psychological development, autonomy-supportive climate, models-based practice

## Abstract

Adults (more than 18 years old) are likely to reproduce the habits that they acquired during childhood and adolescence (from 6 to 16 years old). For that reason, teachers and parents have the responsibility to promote an active and healthy lifestyle in children and adolescents. Even though every school subject should promote healthy activities, Physical Education (PE) is the most important subject to foster well-being habits associated to healthy lifestyle during sport practice and other kinds of active tasks. Indeed, there are many factors that influence the acquisition of healthy habits that should be taken into account when programs and activities are implemented in both educational and extracurricular context. In this sense, psychological and social factors are of utmost importance to achieve optimal experiences for an active and healthy lifestyle. However, due to the myriad of studies analyzing different factors in different contexts, there could be confusion when programs and pedagogical strategies are applied in educational or extracurricular contexts. The objective of this investigation is to analyse the state of art of the psychosocial factors which influence the engagement in physical activities and sport practice. The keywords used in this review were mainly: “Self-Determination Theory,” “(intrinsic) motivation,” “Psychological need satisfaction,” “physical activity and sport engagement,” “Elementary Education,” “Secondary Education,” “Physical Education.” In addition, the Boolean data type “and,” “or,” and “not” were also used. The articles were selected according to the following criteria: (a) peer-reviewed original research published in international journals indexed in JCR or SJR, (b) published in English or Spanish, (c) about psychosocial factors which influence the physical activity and sport engagement, (d) in educational or extracurricular context. Research articles selected were found through Web of Science, Scopus, Google Scholar, SportDiscus (EBSCO-host), ERIC, PubMed, Medline, and PsycInfo databases. It was observed that physical activities and sport practice engagement are closely related to psychological factors. In particularly, intrinsic motivation was able to determine the active participation in any activity, including physical activity and sport practice during the implementation of Small-Sided Games and other kinds of pedagogical strategies (e.g., Pedagogical Models). Motivation was also closely related to flow state. Finally, these variables should be considered in order to organize effective programs to promote an active and healthy lifestyle in Physical Education classes.

## Introduction

Physical inactivity is a serious worldwide problem observed in childhood and adolescence (6–18 years old), as World Health Organization (2016) highlighted in its most recent report. In this sense, it is widely investigated that inactivity is associated with serious physical diseases (e.g., overweight and obesity) and mental disorders (e.g., depression) (Knight, 2012). Although, this population shows a desire to be more active, the external (environmental) factors (e.g., the “screen culture”) significantly influence the final decision to dedicate less time to healthy physical activities (Ennis, 2017).

For that reason, general public policies and specific educational policies from the Administration authorities play a determinant role in the promotion of active and healthy habits alongside the efforts of families and experts (Pate and Dowda, 2019). Regarding to educational context, Physical Education (PE) is the most important subject to achieve this mission due to its unique active and practical frameworks in contrast to the rest of the areas (Kohl and Cook, 2013). The contents of this subject are organized in several disciplines (e.g., adventure education, health-related physical activity components, sport literacy, or teaching dance) depending on the educational curriculum of each country (Kirk et al., 2006). Thus, Simone-Rychen and Hersh-Salganik (2003) proposed the development of all the subjects' contents through the *holistic model of competence* within the Definition and Selection of Competencies framework. Recently, Escalié et al. (2017) emphasized that each piece of content aims to develop a holistic students' development taking into account the *pedagogy of integration* alongside the rest of subjects.

Specifically in the educational context, one of the most established and debated content in PE curriculums around the world is the sport-based and lifestyle programs (Green et al., 2005). Hence, the sport alphabetization or sport literacy in PE is the unique content in the school curriculum aimed to develop the *sport competence*, which is the capacity to deal with a wide range of tactical/technical problems during the sport practice (Kolovelonis and Goudas, 2018). A myriad of research has investigated the best way to meaningfully acquire and develop the sport competence through sport programs using the Pedagogical Models (Haerens et al., 2011), also known as Models-Based Practice (MsBP; Casey, 2014) or Instructional Models (Metzler, 2017).

The MsBP include different pedagogical features to help practitioners to implement sport contents in a contextualized and confident way (Casey and MacPhail, 2018). For this purpose, the MsBP have been classified in several categories according to their final objectives. Hence, the Game-Centered Approach (GCA; Harvey and Jarrett, 2014) is mainly focused on the tactical/technical intelligence of the game, and it includes the Teaching Games for Understanding (TGfU; Bunker and Thorpe, 1982) and its variations around the world; the Sport Education Model (SEM; Siedentop et al., 2019) is dedicated to create an authentic sport experience; the Teaching for Personal and Social Responsibility (TPSR; Hellison, 2011) is focused on facilitating life skills through the sport practice; and finally, the Cooperative Learning (CL; Johnson and Johnson, 1994) aims to develop cooperative performance during the sport practice. In spite of the fact that these are the most implemented models around the world, this is not a complete catalog of them (Casey, 2014). For example, Constraints-Led Approach (CLA; Davids et al., 2005) is also a model of the non-linear pedagogy that aims to develop skill acquisition and motor learning of the whole spectrum of exercise and sport categories (Renshaw et al., 2015).

However, Lund and Tannehill (2010) emphasized that isolated MsBP present several limitations when they are implemented due to the fact that each model is mainly focused on a specific content area (e.g., the tactical/technical elements of the game in the case of GCA). In order to minimize this impact, a recent systematic review proposed the hybridization or combination of two or more models (González-Víllora et al., 2018).

In this context, PE is the ideal subject to promote active and healthy habits, to acquire sport competence as well as to foster active resources for the students' leisure time (Girard et al., 2019). However, according to Perlman (2012a), it is vital to implement well-designed and comprehensive PE programs which (I) take into consideration the elements of the context (e.g., educational content, students or special needs), and which (II) provide students with half of the time of each lessons in moderate-to-vigorous physical activity levels. That is to say, PE does not have intrinsic benefits if it is not adapted to the circumstances of the context where it is going to be implemented. For that reason, it is important to analyse those aspects related to the psychological variables that are definitively able to determine a sense of enthusiasm for learning and improving new skills, and consequently, a sense of engagement for dedicating more time to do physical and sporting activities (Carrasco-Beltrán et al., 2018).

Motivation is a psychosocial process characterized by behaviors that an individual deems vital for his/her personal development (Ryan and Deci, 2000b). These behaviors might change thought time due to the fact that both internal and external factors might affect the personal interests or desires for carrying out a determinant task (Vansteenkiste et al., 2019). The research about motivation in educational contexts is rooted in the Self-Determination Theory (SDT, Deci et al., 1991). Basically, this approach analyses the reasons that students own to engage in certain kinds of activities (Gillison et al., 2019). Otherwise, several approaches have been also proposed to analyse other psychosocial determinants which similarly influence on the motivation of the students, complementing the SDT.

### The Self-Determination Theory (SDT) and the Basic Psychological Needs (BPNs)

The SDT is a complex empirically-based and organismic theoretical framework of the human motivation (Deci et al., 1994; Ryan and Deci, 2017). In this sense, this theory analyses how psychosocial factors influence the human behavior. Specifically in the educational context, the SDT analyses environments and pedagogical factors which influence the students' inherent interest in learning and discovering the world (Deci et al., 1991). Additionally, Ryan and Deci (2017) highlighted that SDT is composed by six mini-theories: the *cognitive evaluation theory, the organismic integration theory, the goal content theory, the relationship motivation theory, the causality orientation theory*, and the *Basic Psychological Needs (BPNs) theory*.

Behaviors and motivation can change over the time, affecting on the individual's performance. For that reason, there is a necessity of categorizing the kinds of motivations that have an impact on the human behavior. Hence, Ryan and Deci (2000a) proposed a continuum of three different motivation constructs depending on the degree of self-determinance: intrinsic motivation, extrinsic motivation and amotivation. In this way, (I) the most self-determined motivation is the *intrinsic motivation*. It is present when people do an activity for inherent and personal reasons as a result of the delight and satisfaction that the practice itself implies. For instance, a student is intrinsically motivated when they enjoy practicing a specific sport because he feel pleasure, and behave in a uninhibited way when they are playing it.

(II) *External motivation* is showed when there are external or environmental factors that condition the people behavior. This construct is divided into four types or levels of regulation. (A) The first level, which is the closest to intrinsic motivation is called *integrated regulation*. It is present when people are aware by the significance of implementing certain behaviors according to the person values. For instance, when a student chose to practice a specific sport because he identified the benefits of practicing it (e.g., learning new technical skills, making friends or being more active) and, in addition, it is congruent with his/her personal values. However, in educational context, it is usual that integrated regulation was not measured (Perlman, 2011; Aelterman et al., 2012; Fernández-Río et al., 2017) due to the fact that this regulation requires a high degree of introspection and relationship with adult self-awareness (Brickell and Chatzisarantis, 2007). (B) The second level is called *identified regulation*. It is present when people's motivation comes from the beliefs that implementing certain behavior is beneficial or important. For example, when it is observed a proactive behavior, defined by the student, to practice certain sport during the schools breaks. (C) The third level is called *introjected regulation*. It is when the pleople's behavior is oriented to avoid a sense of guilty. That is to say, the activity is not accepted as a behavior. For instance, when a non-skilled students are involved in a specific skill-drill game in PE due not to disappoint their peers or teachers/coaches. Finally, (D) *external regulation* is when people practice any kind of activity in order to receive a reward or also to avoid a punishment. It is the least self-determined kind of motivation. For example, when a student always participates in a specific sport in PE, decided by the teacher or the majority of students, trying to avoid a low mark in the final results of the subject (avoidance of punishment).

Finally, (II) *amotivation* is when there is an absence of any kind of motivation in practicing any kind of activities. It is present when people act passively through an activity. For instance, when students are obligated to run around the futsal field during the first 15 min of the PE class as a warm up. In this sense, as Gillison et al. (2013) highlighted, amotivation can cause disruptive behaviors and general disagreement for the activity itself that might produce a reject for practicing similar activities in other contexts (e.g., extracurricular environments).

The Self-Determination Index (SDI; also known as Relative Autonomy Index, RAI; Vallerand, 2007) is a quantitative method that enables researchers and/or other kind of practitioners to determine the total score of the SDT continuum executing the following formula:

$$\begin{array}{lll} SDI\;\approx \;RAI & = & \left(2Intr.\;mot.\right)+Iden.\;reg.\\ -\left(\;\frac{Intro.\;reg.\;+\;Ext.\;reg.\;}{2}\;\right)-\left(2Amo.\right) \end{array}$$

Where *Intr. mot*. is intrinsic motivation, *Iden. reg*. is identified regulation, *Intro. reg*. is introjected regulation; *Ext. reg*. is external regulation; and *Amo*. is amotivation. The mathematical symbol ≈ means approximately equal. Recently, Ünlü (2018) proposed an adjusted of weights in the formula due to the original formula does not take into consideration whether the identified and introjected regulation types are internal and external. For that reason, he proposed to use:

$$\begin{array}{lll} SDIadj\;\approx \;RAIadj & = & mean\;internal\;motivation\\ - & mean\;external\;motivation \end{array}$$

Where the means are calculated using the π weights of the identified and introjected regulations. However, educational studies tend to adapt the original formula to the characteristic of the context (e.g., Perlman, 2011 or Prusak et al., 2004).

On the other hand, it is observed that there are three basic psychological and social nutrients that are able to determine the level of the individual's well-being and its self-determined motivation in the mentioned continuum. Hence, the BPNs comprise three innate and universally psychological needs components that have to be satisfied (and supported) in order to increase the most self-determined motivation: *autonomy* (i.e., sense of control that student interiorized on his/her behavior), *competence* (i.e., sense of mastery or ability that students perceived during a task), and *relatedness* (i.e., regarding the feeling of acceptance, belonging, and unity that the students experience with his/her peers in the same context) (Ryan and Deci, 2000b). According to Vicente et al. (2019), the BPNs are very sensitive to external factors such as the vicarious learning, which can boost or undermine the engagement and motivation to learn new things or skills. Since, the kind of motivation of the SDT are closely related to the BPNs and the external environmental factors, Vallerand (2007) proposed the Hierarchical Model of Motivation in order to relate the BPNs with the SDT continuum. Recently, Prentice et al. (2019) have proposed that the traits of the Whole Trait Theory (i.e., the link between motivational and social-cognitive elements that generate momentary enactments over the time) are an effective way to satisfy the BPNs. For that reason, these authors proposed that this theory are closely related to the SDT.

### The Way to Increase Enjoyment and Adherence to Physical-Sport Activity in PE Programs

A myriad of research in PE and sport context emphasizes that students (or athletes) who perceive higher levels of autonomy, competence and relatedness exteriorize more self-determined forms of regulation and intrinsic motivation (García-Calvo et al., 2010; Vallerand and Lalande, 2011). In this sense, when a student are more engaged in PE, he/she demonstrates more enjoyment, and consequently, exteriorizes a desire to continue playing the sport in his/her leisure time (Browne et al., 2004). This fact has been widely investigated in PE observational studies (Sparks et al., 2017; Navarro-Patón et al., 2018), but also in extracurricular context such as in youth soccer (García-Mas et al., 2010) or among elite sports athletes (Keegan et al., 2014; Thomas and Güllich, 2019).

Hence, self-determined motivation can be promoted among the students designing motivational climates that support the BPNs at PE settings. In this sense, these kind of environments will consequently increase the adherence to practice sport and lifestyle activities beyond the educational context.

However, to our knowledge there is a lack of synthesis that summarize the findings of empirical interventions that aims to demonstrate that innovative MsBP and other pedagogical strategies have the potential to increase the self-determined forms of motivation among students whereas the sport competence are holistically acquired, in contrast to traditional Direct Instruction (DI) approaches, in which technical skill practice are implemented in decontextualized skill-drills forms. In fact, there is just one meta-analysis (Braithwaite et al., 2011) that analyzed the PE motivational climate using the TARGET pedagogical strategy (Epstein, 1989) around the world.

### Research Question, Objectives, and Hypothesis

For all the aforementioned considerations, it is necessary to analyse the state of the art about the positive effects that pedagogical strategies and innovative MsBP applications to the sport literacy PE programs have on the students' psychosocial variables (e.g., self-determined motivation, autonomy, or sense of belonging), which directly influence on the adherence or engagement to active lifestyles.

In this sense, the following research question was formulated: “Are the innovative MsBP and the climate-supported strategies important pedagogical resources that positively impact on the self-determined motivation and the satisfaction of the BPNs to acquire lifelong active, healthy and sporty habits, in contrast to the application of traditional DI approaches in sport literacy at PE context?” Hence, the main objective of the present study was to synthetize the scientific literature findings about the impact of the most important MsBP (i.e., CL, DI, GCA, SEM, and TPSR) as well as supportive-climate strategies (e.g., TARGET) during PE sport literacy content on the students' motivation climate. The second objective of this research was to quantitatively analyse the original studies that determined the impact of the SDI between the MsBP and the traditional DI approach during PE-sport lesson plans.

The first hypothesis states that the implementation of innovative and pedagogical resources such as the MsBP or the TARGET structure are able to (I) increase the students' self-determined motivation and to (II) positive satisfy the BPNs, which directly influence on the adherence of active and sporty lifestyles. The second hypothesis states that the application of MsBP, in contrast to traditional approaches, increases the total students' rates of SDI.

## Methodology

### Systematic Review Protocol

In order to carry out the present systematic review and meta-analysis, the protocol was submitted to PROSPERO database (https://www.crd.york.ac.uk/prospero/) including every relevant information that will be implemented in the systematic review process. In this way, CRD42019125470 is the identification number of the protocol for the present systematic review and meta-analysis.

It was confirmed in PROSPERO that only one first search was carried out previously in the PROSPERO database (in order to corroborate there were not any other registered protocol that investigated the same topic using the same inclusion criteria). After the design of the protocol and its submission on the database were completed, the systematic review process started.

### Search Strategy and Keywords

First of all, an exhaustive and systematic search about original and empirical studies which analyzed the psychosocial factors using MsBP or pedagogical strategies applied in sport or life-style activities programs at PE context was conducted using nine literature database (i.e., Web of Science, SCOPUS, Medline, Google Scholar, SportDiscus, EBSCOhost, ERIC, PsycINFO, and PubMed).

The aforementioned databases were used due to the fact that they comprise PE investigations indexed in Journal Citation Report (JCR) and Scimago Journal Rank (SJR) journals. In addition, the combination of these databases enables to obtain a faithful state of the art of the phenomenon under study using empirical evidence with high-quality standards.

**Figure 1:**
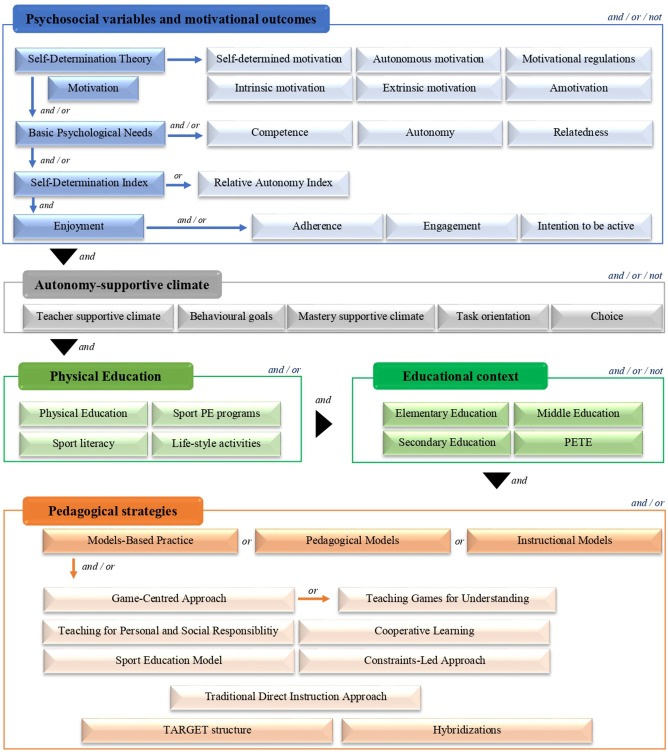
Search equation used during the search.

Figure 1 showed the combination of the keywords and the English Boolean data types (i.e., and, or, not) used in the search equation. In this sense, keywords included important concepts and/or synonyms used in the scientific literature about the psychosocial variables and motivational outcomes (e.g., self-determined motivation, enjoyment, or adherence), the autonomy support climate (e.g., mastery supportive climate or choice), the PE environment (e.g., sport PE programs), the educational context (e.g., Secondary Education), and the pedagogical strategies (e.g., Models-Based Practice, Cooperative Learning, or Sport Education Model) implemented in each primary study.

### Systematic Review and Meta-Analysis Selection Criteria

The articles selected in the present systematic review met the following selection criteria: (I) original research published in peer-reviewed online international journals indexed in JCR or SJR; (II) intervention studies that implemented one or two MsBP (i.e., CL, CLA, GCA, SEM, and TPRS), hybrid MsBP or autonomy-supportive strategies (e.g., TARGET) in PE sport units; (III) research that implemented one or more sports content (e.g., soccer, basketball, or track and field) or life-style activities (e.g., walking) intervention studies about the impact of the SDT or the BPNs satisfaction outcomes; (IV) research conducted in a PE or Physical Education Teacher Education (PETE) context; (V) original studies that included quantitative and/or qualitative designs and outcomes; and (VI) research published in English or Spanish, which are the main languages used in MsBP interventions.

In fact, the exclusion criteria included: (I) observational studies, (II) not indexed in JCR or SJR journal, (III) intervention studies published in books, thesis or conference proceedings; and (IV) opinion or pedagogical articles. When articles do not reported important methodological procedures (e.g., sport program implemented, country, or allocation of groups) the protocol of this work established to contact to the correspondence author. If the author do not respond, the article were excluded at the third level of analysis.

On the other hand, the quantitative articles selected for the meta-analysis met the following selection criteria: (I) (quantitative) studies which compare one or more MsBP with the traditional DI or skill-based approach or the comparison of two or more autonomous motivation climate with the traditional DI or skill-based approach; (II) research which included the SDT components or SDI outcomes.

### Search Process, Data Extraction, and Use of Software

The search process was divided into four phrases or levels. The first one is concerned to the initial search on the databases using the aforementioned search equation, adapting it for each database. The second phrase is regarding the classification of the articles by their outcomes (e.g., findings about the satisfaction of the psychological needs or about the task- and ego-orientation), excluding those which do not fit the selection criteria. For this mission, the title, abstract, and keywords were analyzed. In this phrase, duplicated articles from different database were eliminated. The third phrase consisted of a deeper analysis of the methodology and discussion of every potential article. Finally, in the fourth level, those quantitative articles that reported the SDI from the comparison of two groups implementing MsBP or autonomous support climate and traditional DI approach, where included in an extra database for the meta-analysis.

**Figure 2:**
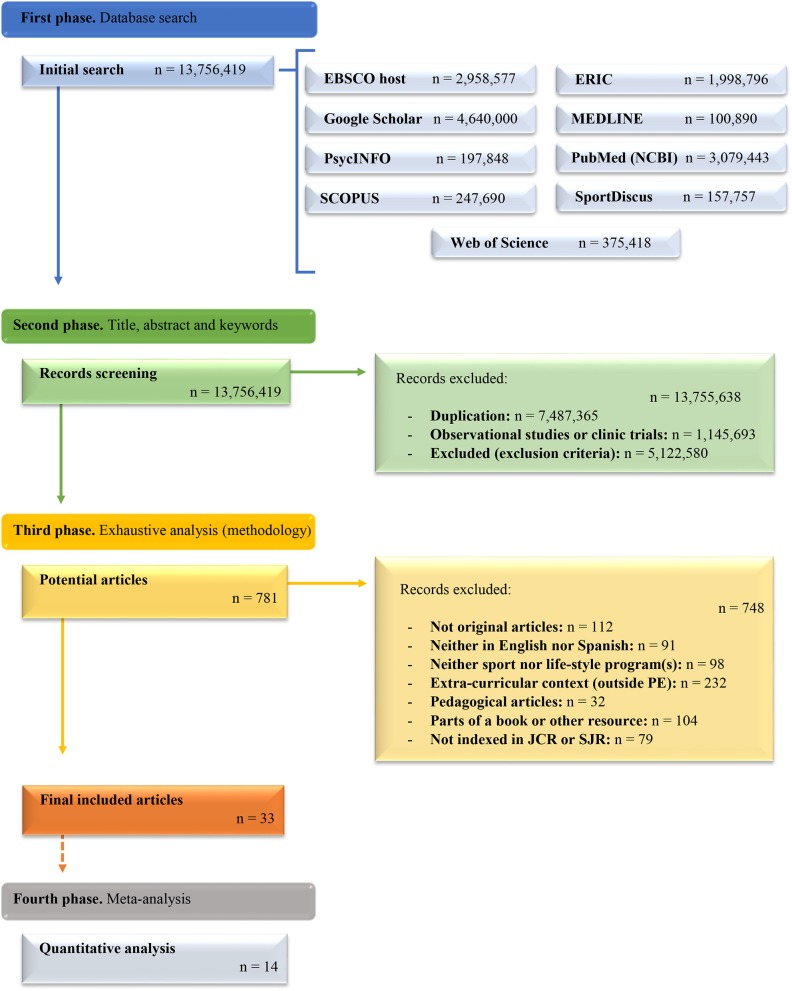
Flow diagram of the phases of the systematic search, screening, and analysis process.

Hence, as it is showed in Figure 2, the initial search comprises 13,756,419. After the second phase 781 were considered for an exhaustive analysis. Finally, 33 articles were firstly considered in the systematic review. In addition, from this number, 14 articles had fitted the meta-analysis inclusion criteria and were retrieved for being meta-analyzed.

For the purpose of this systematic review and meta-analysis, reference manager software Mendeley™ and meta-analysis software Comprehensive Meta-Analysis™ were used. In addition, quality analysis of the final included studies were evaluated using three risks of bias *ad hoc* instruments: the *Checklist for Measuring Study Quality* (Downs and Black, 1998) to assess both randomized and non-randomized studies, the *AXIS Appraisal Tool* (Downes et al., 2016) to assess the quality of cross-sectional studies, and finally, the *Cochrane Ris of Bias 2.0*. (Higgins et al., 2016) adapting several items by the *Strengthening the Reporting of Observation Studies statements* (von-Elm et al., 2008). Additionally, in order to obtain an overall quality score for each study, an exhaustive analysis were carried out based on the systematic review procedure showed in González-Víllora et al. (2018). Finally, the quality of the systematic review and meta-analysis was evaluated using the *Preferred Reporting Items for Systematic Reviews and Meta-Analyses* (PRISMA; Moher et al., 2009).

### Meta-Analysis Procedure

The meta-analysis was executed with 14 quantitative studies from the total pool of articles included in the systematic review (*n* = 33; see Figure 1). In this case, the objective of the meta-analysis was to quantitatively synthesize the findings about the SDI among the studies which carried out an original analysis between the traditional DI approach and the most widely used MsBP at an educational context (i.e., CL, CLA, GCA, SEM, and TPRS). For this mission, the analyses were executed using the Comprehensive Meta-Analysis software™ (CMA™; Lipsey and Wilson, 2001).

In this context, due to the fact that fixed-effect models only calculate the error of the variation in the final analysis influenced by the sample size (Cooper, 2017), random-effects models are proposed because the effect size variation between studies assumes both true-random variance and sampling error from each study (Koutsimani et al., 2019). However, in the present study, both effect models were reported, including the weighted d-index. Additionally, the Cohen's effect size was calculated for every result based on the criteria of Hopkins et al. (2009), where the effect sizes were considered as trivial (<0.2), small (0.2–0.59), moderate (0.6–1.19), large (1.20–2.00), very large (2.00–3.99), and extremely large (>4.00).

Finally, the analysis was grouped by different subgroups based on the MsBP (i.e., CL, DI, GCA, Hybrid models and SEM) and the specific pedagogical strategies (i.e., TARGET structure and autonomy-supportive climate into traditional DI lessons). In this sense, the Study-Level Measure of Effect (*I*^2^) was calculated. This statistic informs about the proportion of the total variance in the effect sizes due to the variance among the studies. According to Cooper (2017), the *I*^2^ statistics above 75% implicate significant heterogeneity. The statistical significance was set up at *p* < 0.050 (95% confidence interval).

## Results

The results were divided into two sections. In the first-one (section *Systematic review findings*), the synthesis of the 33 original articles is presented. In the second-one (section *Meta-analysis findings*), the meta-analysis results of the 14 quantitative research are shown.

### Systematic Review Findings

Table 1 shows the main important findings of each selected article that meets the selection criteria established in the previous section. In order to facilitate the interpretation of the results, the most important and/or relevant information of each article was classified into “Author(s) and year,” “Program and content applied,” “Aim of the investigation,” “Intervention contextualization,” “Methodology,” which was divided into “Instruments” and “Variables (measured by the instruments),” and finally the “Main outcomes.”

**Table 1:** Synthesis of the investigations about psychosocial outcomes in PE sport programs using MsBP and/or autonomy support.

**References**	**Program (Content)**	**Aims**	**Intervention contextualization**	**Methodology**		**Main outcomes**
				**Instruments**	**Variables**	
Clarke and Quill (2003)	PE. SEM. (Netball, soccer, sports acrobatics, and athletics)	To report the benefits of the SEM on students' motivation, involvement in practice and leadership.	England (UK). Two mixed-sex and ability classes of 8th grade (secondary education). Double (120 min) and single (60 min) classes per week during the PE course.	Qualitative; longitudinal design		Students who took the more skilled responsibilities in the model became more motivated, demonstrating a strong sense of ownership. Additionally, they enjoyed taking different roles.
				Participant observation	Field diary notes and teacher-research's diary	
				Semi-structured interviews (during the intervention)	Students perceptions	
O'Donovan (2003)	PE. Normal PE program followed by SEM. (Not reported)	To explore the effects of promoting team affiliation on social goals.	England (UK). 68 7th grade (secondary education) students. Two classes per week.	Qualitative; ethnographic design		Although, no noticeable changes in participation levels were noted, social goals were an important determinant of motivation and participation in PE.
				Participant observation	Field diary notes and video-recorded session	
				Unstructured interviews	Whole-class interview/forum	
Browne et al. (2004)	PE−2 groups. Trad. DI and SEM. (Rugby union)	To compare the effect on students' learning, enjoyment and affect between two MsBP.	Australia. 53 8th grade (secondary education) female students grouped into DI group (*n* = 26) and SEM group (*n* = 27). 10 lessons of two weekly 45-min sessions.	Mixed study; quasi-experimental design		The sense of belonging and responsibility were features that increased greater levels of autonomy at the SEM implementation. In addition, the perception of greater autonomy and organization were also observed in the SEM group.
				Declarative rugby assessment *ad hoc*	Laws and rules of the sport	
				Student self-assessment *ad hoc*	Procedural self- evaluation items	
				Teacher evaluation of skills *ad hoc*	Procedural teacher evaluation	
				Semi-structured interview	*n* = 16; enjoyment skills and affect	
Prusak et al. (2004)*	PE−2 groups. No-choice DI and TARGET strategy unit with choices. (Walking unit of instruction)	To determine students' motivational responses between autonomy and non-autonomy-supportive contexts.	USA. 42 7th and 8th grade (secondary education) students classified into DI group (*n* = 21) and TARGET group (*n* = 21). 10 sessions.	Quantitative; quasi-experimental design		Providing a free selection of activities, students experienced an increase of their situational and intrinsic motivation in contrast to the group which had to participate in imposing activities and was not autonomy-supported.
				SIMS *ad hoc* (Guay et al., 2000)	SD index (RAI) and situational motivation	
				SMSPE *ad hoc* (Briere et al., 1995)	Intrinsic motivation, extrinsic motivation, and amotivation	
Wallhead and Ntoumanis (2004)*	PE−2 groups. Trad. DI and SEM. (Basketball)	To analyse the effects of a SEM and DI interventions in fostering students' enjoyment, as well as perceived and autonomy competence.	England (UK). 51 10th grade (secondary education) students (14.3 ± 0.48), grouped into DI (*n* = 25) and SEM groups (*n* = 26). 8 lessons of one weekly 60-min (50-min real practice) session.	Quantitative; quasi-experimental design		The structure of the SEM is very similar to the TARGET strategies. Indeed, the SEM intervention facilitated the perception of task-involving climate. Additionally, perceived autonomy had a positive effect on student motivational outcome. SEM also showed better results in enjoyment and perceived efforts in contrast to the traditional approach.
				IMI (McAuley et al., 1989)	Enjoyment, effort, and perceived competence	
				TEOSQ (Duda and Nicholls, 1992)	Ego and task goal orientation	
				ASRQ *ad hoc* (Ryan and Connell, 1989) and AMS *ad hoc* (Vallerand et al., 1992)	Different degrees of perceived autonomy	
				LAPOPECQ (Papaioannou, 1995)	Ego- and task- involving climate	
				CBAS (Smoll and Schutz, 1990)	Codification of teacher behavior	
Hastie and Sinelnikov (2006)	PE. SEM giving special attention to the TARGET strategy. (Basketball)	To examine the students' participation and perception of an innovative SEM.	Russia. 37 6th grade (primary education) students. 18 lessons of three weekly 40-min sessions.	Mixed study; quasi-experimental design		Traditional PE in Russia had been following the DI approach. In this context, the implementation of SEM produced an increase of the students' involvement, autonomy, enjoyment, and engagement throughout the whole season.
				Systematic observation and BEST (Sharpe and Koperwas, 1999)	Teacher behavior and students lesson participation	
				PESS (Mohr et al., 2003)	SEM components and features	
				Semi-structured interviews	*n* = 4; students' perceptions	
Mandigo et al. (2008)	PE. TASG. (Target, Striking, Net/Wall, and Invasion games)	To (I) investigate students' motivational experience across different sports, and to (II) compare the gender differences.	Canada. 759 students from 4th to 7th grade (primary education), divided into 9 classes for the Target unit, 11 for the Striking unit, 7 for the Net/Wall unit, and 10 for the Invasion games unit.	Quantitative; quasi-experimental design		Lower impact on students' motivation at the Invasion games unit was observed because it is the most tactical-complex category. The use of TASG is an effective way to foster students' intrinsic motivation.
				IMI *ad hoc* (McAuley et al., 1989) including open-ended responses	Perceptions of the BPNs and intrinsic motivation	
				CPOCI (Mandigo and Sheppard, 2003)	Perception of the optimal challenge	
Gray et al. (2009)	PE−2 groups. Trad. DI and TIG using the TARGET strategy. (Basketball)	To determine the teacher behaviors and the students' motivational climate across two models using the TARGET structure.	Scotland (UK). 51 8th grade (secondary education) students (12.5 ± 0.30), grouped into DI (*n* = 25) and TIG-TARGET groups (*n* = 27), including the two teachers. 5 lessons of one weekly session from 60 to 80 min.	Mixed study; quasi-experimental des		The TIG group teacher showed more mastery behavior. On the contrary, the DI lessons negatively affected the pupil feeling of autonomy, enjoyment effort, and learning based on problem-solving or cooperative context, as it was applied in the TIG.
				Video recording data BEST (Sharpe and Koperwas, 1999)	Effectiveness of application of TARGET	
				Semi-structured teacher	Teachers' experience	
				Semi-structured student interviews and/or focus group	*n* = 4; students' learning experience	
Lonsdale et al. (2009)	PE. Trad. DI including free-choice periods. (Basketball)	To compare the relationship between students' SD motivation and PA level during teacher structured part of the sessions, and free-choice portion of them.	Hong Kong (China). 296 female and 232 male 10th grade (secondary education) students (15.78 ± 0.91 years). 18 lessons of 40 min, divided in 20 min of structured lessons led by the teacher and 20 min of free-choice activity.	Quantitative; quasi-experimental design		Students' motivation were related to high levels of steps in both structured and free-choice part of the lessons. Besides, need-supportive contexts were also related to greater self-determined motivation. It is recommend to integrate free-choice periods into PE.
				SIMS (Guay et al., 2000) Yamax Digi-Walker DW-700™ pedometers	SD index (RAI) Students' steps per minutes	
Spittle and Byrne (2009)	PE−2 groups. Trad. DI and SEM. (Soccer, hockey, and football codes)	To (I) compare the impact of two models on the students' intrinsic and/or extrinsic motivation, goal orientation and perceived motivational climate.	Australia. 115 8th grade (secondary education) students grouped into DI (*n* = 74) and SEM groups (*n* = 41). 10 lessons of one weekly session.	Quantitative; quasi-experimental design		Although. there was no significant difference in enjoyment and perceived effort between both models; perceived competence, task orientation, and mastery climate are significantly higher in the SEM group in contrast to the DI group. For that reason, SEM enhance student motivation.
				IMI (McAuley et al., 1989)	Interest/enjoyment, effort/importance, pressure/tension and perceived competence	
				TEOSQ (Duda and Nicholls, 1992)	Ego and task goal orientation	
				PMCSQ (Walling et al., 1993)	Performance and mastery climate	
Jones et al. (2010)	PE−2 groups. Trad. DI and TGfU. (Invasion games)	To determine the students' intrinsic motivation between the implementation of two models.	England (UK). 202 7th−9th grades (Key Stage three) students and their two teachers. 6 weeks.	Quantitative; quasi-experimental design		Students from the TGfU group showed significantly greater levels of intrinsic motivation. Enjoyment can be engaged using TGfU.
				IMI *ad hoc* (McAuley et al., 1989)	Enjoyment, Pressure/tension, effort, choice and value/usefulness	
Perlman (2010)	PE−2 groups. Trad. DI and SEM. (Basketball, Volleyball, soccer, and lacrosse)	To investigate the affect and needs satisfaction of amotivated students using the SEM and the DI approaches.	USA. 78 9th−12th grades amotivated students from a pool of 1,176, divided into DI (*n* = 16 classes of 40 students) and SEM groups (*n* = 16 classes of 38 students). 15 lessons of three/four weekly 60-min sessions.	Quantitative; quasi-experimental design		The SEM students showed significantly higher levels of enjoyment and relatedness satisfaction, rather than DI students. SEM features such as peer leadership enable more students' engagement into their learning experiences.
				SRQ-PE *ad hoc* and AMS-PE *ad hoc* (Goudas et al., 1994)	Identification of amotivated students	
				IMI *ad hoc* (McAuley et al., 1989)	Enjoyment subscale	
				BPNS-PE *ad hoc* (Ntoumanis, 2005)	BPNs components	
Perlman and Goc-Karp (2010)	PE. SEM. (Flag football and soccer)	To understand the psychosocial variables related to the SDT in a class using the SEM.	USA. 24 secondary education students. Two seasons of three weekly 72-min sessions.	Qualitative; case of study		It was confirmed that the psychosocial needs of both students and teachers could be supported by implementing SEM.
				Interviews	Students and teacher perceptions	
				Field notes	Students and teacher behaviors	
González-Cutre et al. (2011)*	PE−2 groups. Trad. DI and TARGET strategy unit. (Invasion game and sport acrobatics)	To compare the effects of the task-involving climate, 2 × 2 achievement goals and the self-determined motivation by means of a TARGET unit.	Spain. 46 8th grade (secondary education) students (13.39 ± 0.57) divided into DI group (*n* = 20) and TARGET group (*n* = 26). 26 lessons of two weekly 50-min sessions.	Quantitative; quasi-experimental design		It was observed that the TARGET-group students achieved more self-determined motivation, in contrast to the traditional-group students. Hence, the mastery-approach can be supported by programs which priories the students' effort and personal growth. This fact, alongside other psychological variables, can determine the amount and time of extracurricular physical and sport activities practiced by the students.
				PMCSQ-2 *ad hoc* (Newton et al., 2000)	Ego and task-involving climate	
				PSPP *ad hoc* (Fox and Corbin, 1989)	Perceived competence	
				2 × 2 -AGF *ad hoc* (Elliot and McGregor, 2001)	Achievement-goals components	
				SGS-PE *ad hoc* (Guan et al., 2006)	Responsibility and relationship goals.	
				PLOCS *ad hoc* (Goudas et al., 1994)	SDT components	
				DFS-2 *ad hoc* (Jackson and Eklund, 2002)	Flow state	
Perlman (2011)*	PE−2 groups. Trad. DI and SEM. (Volleyball)	To examine the impact of a SEM season on students' self-determination and BPNs variables.	USA. 182 9th grade (secondary education) students (14.3 ± 0.48), grouped into DI (*n* = 88) and SEM groups (*n* = 94). 20 lessons of four weekly 60-min sessions.	Quantitative; quasi-experimental design		SEM students were significantly more self-motivated and reported higher levels of relatedness in contrast to DI students. This fact allows social connections between peers and students.
				SMS *ad hoc* (Pelletier et al., 1995)	SDT components, including Intrinsic motivation to know and SDI.	
				BPNS-PE (Ntoumanis, 2005)	BPNs components	
Perlman (2012b)*	PETE−2 groups. Trad. DI and SEM.	To assess the influence of using the SEM on the teachers' autonomous instruction.	Australia. 50 pre-service secondary PE teachers randomly assigned to a traditional DI group (*n* = 25) and SEM group (*n* = 25). 15 lessons of 60-min sessions during 16 weeks.	Quantitative; quasi-experimental design		Pre-service teachers whom participated in the SEM group showed better autonomous behaviors in contrast to the traditional model group. Significant changes in perception of autonomy-support were found in SEM in contrast to the traditional group.
				Coding and observational autonomous instruction method (Sarrazin et al., 2006)	Teacher's instruction style (autonomous, controlling, or neutral)	
				LCQ (Williams and Deci, 1996)	Perception of autonomy-support	
				SMS *ad hoc* (Pelletier et al., 1995)	SDT components	
Gillison et al. (2013)	PE−4 groups. Trad. DI using different autonomy- or controlling- supportive climate instructions. (Fitness-based circuits)	To evaluate the students' motivational level and intention to be active on different autonomy- and controlling-supportive climate lessons of fitness.	England (UK). 592 9th grade (secondary education) students. One experimental lesson. After the teacher demonstration of each activity, the lesson began with a warm-up, followed by a circuit of 10 fitness activities with 30 s of duration, including 2 min of break at the middle of the lesson.	Quantitative; quasi-experimental design		On the one hand, the students whom were autonomously supported by their teacher significantly increased their self-determined motivation and their positive intention to exercise in contrast to those students whom received a controlling supportive climate. On the other hand, this study highlighted the difficulty of manipulating social and goal contexts to engage active students.
				PLOCS (Goudas et al., 1994) and SIMS (Guay et al., 2000)	SDT and behavioral regulations components	
				IMI *ad hoc* (McAuley et al., 1989)	Interest, effort, and enjoyment of the lesson and activity value	
				LCQ *ad hoc* (Williams and Deci, 1996)	Perception of autonomy support	
				EFI (Gauvin and Rejeski, 1993)	Change in mood and vitality after the activity	
Amado et al. (2014)	PE−2 groups. Trad. DI and multi-dimensional intervention. (Dance)	To analyse the students' self-determined motivation as well as the satisfaction of the BPNs through dance.	Spain. 47 10th grade (secondary education) students (14.84 ± 0.84 years), divided into DI group (*n* = 27) and multi-dimensional intervention group (*n* = 20). 12 lessons of two weekly 50-min sessions.	Quantitative; quasi-experimental design		A significant difference was observed in the need for autonomy among participants in the multi-dimensional intervention. This kind of programs, focused on supporting the BPNs, shows a positive effect among children adherence to physical activity.
				BPNMS *ad hoc* (Vlachopoulos and Michailidou, 2006)	BPNs components	
				MDCEQ *ad hoc* (Amado et al., 2012)	SDT components except integrated and introjected regulation	
Báguena-Mainar et al. (2014)*	PE-2 groups. Trad. DI and TGfU with TARGET. (Volleyball)	To investigate the impact of a GCA program using the TARGET strategy in the students' motivation.	Spain. 61 10th grade (secondary education) students (15.88 ± 0.84) grouped in DI (*n* = 20) and TGfU with TARGET structure (*n* = 41) group. 10 lessons of two weekly 50-min sessions.	Quantitative; quasi-experimental design		The use of TGfU alongside the TARGET strategy significantly fostered the students' task-orientation and the autonomy support, engaging them to be more active, in contrast to traditional PE frameworks. Controlling environmental models (i.e., DI) are likely to produce a decrease in the students' enjoyment.
				PPECCS *ad hoc* (Biddle et al., 1995)	Ego and task involving climate	
				ASCQ *ad hoc* (Conroy and Coatsworth, 2007)	Autonomous behavior and students' opinion	
				BPNES *ad hoc* (Vlachopoulos and Michailidou, 2006)	BPNs components	
				SIMS-14 *ad hoc* (Guay et al., 2000)	SDT components except integrated and introjected	
Goodyear et al. (2014)	PE. CL. (Basketball)	To analyse the implementation of a model to increase responsibility for the students' self-learning and engagement with the PE contents.	England (UK). Two classes of 10th grade (secondary education) female students. Eight lessons for a minimum of 2 h per week.	Qualitative; quasi-experimental design		CL (with the use of flip cameras during the unit) was reported as a beneficial model to empower female students' responsibility, cooperation and collaboration with their peers. Hence, students' engagement is enhanced with the CL approach.
				Reflexible teacher journal, PLTA (Casey et al., 2009) and videorecordings produced by the students	Evaluation of CL learning elements. Students' behaviors, participation, and engagement.	
				Student team semi-structured interviews	Students' participation	
Hastie et al. (2014)	PE. SEM using the TARGET strategy. (Handball)	To analyse the implementation of SEM that emphasizes the mastery-involving climate among students' motivation.	USA. 21 secondary education male students and one teacher. 12 lessons of one weekly of 90-min sessions.	Quantitative; quasi-experimental design		SEM features can be oriented to a mastery-oriented climate throughout the season. In this context, the TARGET structure was an additive to the students' motivation and mastery-oriented climate.
				Videorecording sessions and BEST (Sharpe and Koperwas, 1999)	Teaching behavior related to motivational climate	
				TEGQ (Papaioannou et al., 2007)	Motivational climate students' perception	
Smith et al. (2014)	PE−2 groups. Trad. DI and TGM. (Netball and football for girls; plus rugby and football for boys)	To examine the levels of moderate-to-vigorous physical activity and the self-determined motivation among female and male students using two models.	England (UK). 72 7th grade (secondary education) students (11.31 ± 0.45) from two schools, divided into DI groups (girls class = 17, boys class = 19) and TGM groups (girls class = 13, boys class = 23). 12 lessons for each model.	Quantitative; quasi-experimental design		Although physical activity levels were higher in the female TGM class; it is significantly higher in the male TGM class, in contrast to both DI and TGM classes. However, there were no significant differences in self-determined motivation between TGM and DI.
				SOFIT (McKenzie, 2012) and RT2™ triaxial acceleromenter	Quantification of the activity level	
				Self-Determination Questionnaire (Standage et al., 2005)	Intrinsic motivation and BPNs components	
				IMI *ad hoc* (McAuley et al., 1989)	Enjoyment subscale	
Wallhead et al. (2014)*	PE−2 groups. Multi-activity DI and SEM. (Floor hockey, volleyball, handball, basketball, badminton, cooperative games, and soccer)	To investigate the impact of the SEM using different sports on the students' motivation, and their influence on the leisure-time physical activity.	USA. 568 secondary education students (14.75 ± 0.48 years) from two schools. 25 lessons of SEM (first school) and from four- to nine-block lessons of DI (second school). SEM benchmark observation instrument were used (Ko et al., 2006).	Mixed study; quasi-experimental design		SEM students reported greater interest due to an increase of enjoyment and self-determined motivation, in contrast to multi-activity DI program students. However, the results showed a small increase over time in the intention to practice leisure-time physical activity among SEM students.
				PLOCS (Goudas et al., 1994)	SDT components except integrated regulation	
				AMS-PE *ad hoc* (Goudas et al., 1994)	Amotivation subscale	
				IMI *ad hoc* (McAuley et al., 1989)	Enjoyment subscale	
				PAIS (Ajzen, 2003) and LTEQ (Godin and Shephard, 1985)	Intention to be physically active	
Chatzipanteli et al. (2015)*	PE−2 groups. Trad. DI and teaching style program. (Basketball, volleyball, soccer, fitness, track and field, and gymnastics)	To compare the effects of different student-centered teaching styles on the student self-regulation, motivation and lesson satisfaction from a PE program.	Greece. 601 7th grade students (secondary education), assigned into Trad. DI group (*n* = 285) and supportive climate group using different teaching styles (*n* = 316). 38 lesson of three weekly 45-min sessions.	Quantitative; quasi-experimental design		Student-centered teaching style group showed significantly higher marks on the metacognitive outcomes. Additionally, this group also reported significantly higher levels of self-determined motivation, in contrast to the traditional one.
				MPPEQ *ad hoc* (Theodosiou and Papaioannou, 2006)	Students' metacognition about the sports	
				SIMS *ad hoc* (Guay et al., 2000)	SDT components except integrated and integrated regulation.	
				LSSCL *ad hoc* (Duda and Nicholls, 1992)	Lesson satisfaction	
Moy et al. (2016)	PETE−2 groups. Trad. DI and CLA. (Hurdles unit)	To corroborate that the CLA model can effectively orient students toward the positive satisfaction of the three BPNs.	Australia. 54 second-year pre-service PETE students, divided into two groups who experience DI firstly and CLA secondly; and vice versa. 2 lessons of 50-min.	Quantitative; quasi-experimental design		The BPNs, effort and enjoyment were significantly better in both CLA groups, reporting more self-determined motivated behaviors than in both DI groups.
				IMI *ad hoc* (McAuley et al., 1989)	Enjoyment, effort, and BPNs components.	
Chang et al. (2016)*	PE−2 groups. Trad. DI and Trad. DI using autonomy-support strategy. (PE program of multiple sports)	To assess the impact of changing the teaching style in a traditional PE program (including running, jumping, vaulting boxes, badminton, Chinese yo-yo, and basketball) on students' motivation.	Taiwan. 126 6th grade (elementary education) students, assigned to DI (*n* = 65) and autonomy-supportive groups (*n* = 61). 12 lessons of two weekly 40-min sessions. Each sport was taught twice per week.	Quantitative; quasi-experimental design		PE lessons manipulated by supporting students' autonomy reported an increase of their intrinsic motivation. In this sense, the students from the autonomy-supportive group showed greater levels of perceived autonomy when students had more choices in selecting partners, contents, and/or learning tasks.
				Perceived teacher autonomy questionnaire *ad hoc* (Standage et al., 2006)	Students' perceived autonomy by the teacher	
				Perceived autonomy questionnaire *ad hoc* (Standage et al., 2006)	Students' perceived autonomy in PE	
				Self-determined motivation scale *ad hoc* Ntoumanis (2001)	SDT components except integrated regulation	
Cuevas et al. (2016)*	PE−2 groups. Trad. DI and SEM. (Volleyball)	To compare the effect of the traditional model and the SEM on the students' motivational outcomes.	Spain. 86 10th grade (secondary education) students (15.65 ± 0.78 years) grouped into DI team (*n* = 43) and SEM team (*n* = 13). 19 lessons of two weekly 55-min sessions.	Quantitative; quasi-experimental design		Although, it was observed slight improvements in the SDI and identified regulation among SEM students, intrinsic motivation significantly improved in contrast to DI students. Otherwise, no changes were observed in the perceptions of the thwarting autonomy and relatedness at SEM students.
				QEMPE (Sánchez-Oliva et al., 2012)	SD components expect integrated regulation	
				PNTS *ad hoc* (Bartholomew et al., 2011)	Thwarting of autonomy, competence, and relatedness	
				SSI *ad hoc* (Balaguer et al., 1997)	Satisfaction-enjoyment and boredom	
				IPAS *ad hoc* (Hein et al., 2004)	Intention to be physically active	
Burgueño et al. (2017)*	PE−2 groups. Trad. DI and SEM. (Basketball)	To compare the impact of the students' motivational regulation between the implementation of traditional DI unit and SEM.	Spain. 44 11th grade (secondary education) students (16.32 ± 0.57 years) assigned to DI team (*n* = 22) and CL team (*n* = 22). 12 lessons of two weekly 55-min sessions.	Mixed study; quasi-experimental design		The SEM season significantly produced an increase of the intrinsic motivation and identified regulation, including a decrease of external regulation and amotivation in contrast to the DI group.
				SMS *ad hoc* (Guay et al., 2000)	SDI; Identified motivation, identified regulation, external regulation, and amotivation	
Fernández-Río et al. (2017)**	PE−2 groups. Trad. DI and CL. (Cooperative physical challenges *ad hoc*, Coop fitness *ad hoc*, and Cooperative parkour *ad hoc*)	To (I) determine the impact of students' motivation across the CL approach, (II) assessing the students' perception, as well as (III) feelings and thoughts about this model.	Spain. 249 from 8th to 11th grades (secondary education) students (13.41 ± 1.25 years) and their four teachers assigned to DI (*n* = 112) and CL groups (*n* = 137). 16 weeks of 2 h every week. Each unit (Cooperative physical challenges, Coop fitness and Cooperative parkour *ad hoc*) has a duration of 10 sessions.	Mixed study; quasi-experimental design		It is demonstrated that the application of the CL approach increases the students' most self-determined kinds of motivation. Indeed, students' perceptions showed the ideas of cooperation, relatedness, enjoyment and novelty, which produced a positive impact during the CL intervention.
				PLOCS *ad hoc* (Goudas et al., 1994) PMCSQ-2 *ad hoc* (Newton et al., 2000)Students' perceptions open-ended question (Qualitative approach)	SDT components except integrated regulation Cooperative learning subscale Students' perceptions about the CL experience particularly, and the whole experience generally.	
Gil-Arias et al. (2017)*	PE−2 groups. Trad. DI and Hybrid TGfU/SEM. (Volleyball and Ultimate Frisbee™)	To assess the effect between a hybrid TGfU/SEM and a traditional DI unit on students' self-determined motivation, as well as on their adherence in PE programs.	Spain. 55 9th−10th grades (secondary education) students (15.45 ± 0.41), divided into group A (*n* = 27; Hybrid firstly and DI secondly) and group B (*n* = 28; DI firstly and Hybrid secondly). 16 lessons of two weekly 50-min sessions. Each model lasted 8 lessons.	Mixed study; quasi-experimental design		When students participated in the Hybrid TGfU/SEM unit, they showed greater levels of autonomy and competence, in contrast to DI units. In addition, group A (i.e., hybrid unit first) obtained higher scores on self-determined motivational variables than group B (i.e., DI unit first).
				PLOCS *ad hoc* (Goudas et al., 1994)	Autonomous motivation and SDT components	
				BPNES *ad hoc* (Vlachopoulos and Michailidou, 2006)	BPNs components	
				EBSS *ad hoc* (Duda and Nicholls, 1992)	Enjoyment	
				IPAS (Arias-Estero et al., 2013)	Intention to be physically active	
Harvey et al. (2017)	PE. CGA-TGM. (Basketball)	To examine the students' perceptions of BPNs and self-determined motivation applying a CGA-TGM unit.	USA. 94 elementary students and 79 middle school students. 33 lesson of one weekly 40-min sessions at elementary school, and 32 lessons of four weekly 43–49 min at middle school. Application of the model benchmark to ensure an optimal implementation of the models.	Quantitative; quasi-experimental design		This model showed a significant increase of the relatedness perception in elementary and middle students. Indeed, the implementation of this model enables students to first learn the tactical aspects of the game in a contextualized situation using modified and/or Small-Sided Games. Besides, longer-term TGM implementation enhances the reduction of controlling teacher behaviors.
				BPNs and SDT questionnaire protocol (Standage et al., 2005)	Three BPNs components and the SDT components except integrated regulation	
				SOFIT (McKenzie, 2012)	Lesson context evaluation	
				WVUTES (Hawkins and Wiegand, 1989)	11 teacher behavior patterns evaluation (e.g., positive feedback or physical guidance)	
Chiva-Bartoll et al. (2018)*	PE-2 groups. Trad. DI and hybrid CL/TGfU. (Handball)	To examine and compare the students' motivational climate between a hybrid CL/TGfU model and a traditional approach.	Spain. 96 10th grade (secondary education) students (15.00 ± 0.7 years), divided into 31 students in the traditional approach group, and 65 in the hybrid approach group. 8 lessons of two weekly 55–60-min sessions.	Quantitative; quasi-experimental design		The evolution of the motivational climate did not show significant differences among groups. However, the hybrid model contributed to the increase of task-involvement, as well as the decrease of ego-involvement.
				PMCSQ-2 (Newton et al., 2000)	Motivational climate divided into (I) task-involvement and (II) ego-involvement subscales.	
Vazou et al. (2019)	PE-2 groups. Trad. fitness unit and BPNs supportive- climate fitness-practice lesson (Fitness: running, curl-ups, and push-ups).	To investigate the motivational factors that could be fostered by the PE teacher introducing supportive-climate elements in PE fitness-practice lessons.	USA. 148 4th−6th grade (elementary education) students (10.39 ± 0.98 years) divided into traditional and supportive-climate groups. Two lessons (one for each group) of 30 min.	Quantitative; quasi-experimental design		Since physical fitness could be considered an unenjoyable activity, the implementation of play-like elements in the supportive climate group, prevented the declined of affective valence, and increase of enjoyment, in contrast to the traditional fitness group.
				SenseWear Armband™ monitor	Physical activity level	
				FS (Hardy and Rejeski, 1989)	Affective valence	
				S-PACES (Paxton et al., 2008)	Enjoyment	
				AFSS (Reeve and Sickenius, 1994)	BPNs components	

*In order of appearance: PE, Physical Education; SEM, Sport Education Model; UK, United Kingdom; Trad. DI, Traditional Direct Instruction; MsBP, Models-Based Practice; TARGET, Task, Authority, Recognition, Grouping, Evaluation, and Time; USA, United States of America; SIMS, Situational Intrinsic Motivation Scale; SD, Self-Determined; RAI, Relative Autonomy Index; SMSPE, Sport Motivation Scale for Physical Education; IMI, Intrinsic Motivation Inventory; TEOSQ, Task and Ego Orientation in Sport Questionnaire; ASRQ, Academic Self-Regulation Questionnaire; AMS, Academic Motivation Scale; LAPOPECQ, Learning and Performance Orientations in Physical Education Classes Questionnaire; CBAS, Coach Behavior Assessment System; BEST, Behavioral Evaluation Strategies and Taxonomies; PESS, Physical Education Season Survey; TASG, Teaching-Autonomy-Supportive Games; BPNs, Basic Psychological Needs; CPOCI, Children's Perception of Optimal Challenge Inventory; TIG, Team Invasion Games; PA, Physical Activity; PMCSQ, Perceived Motivational Climate in Sport Questionnaire; TGfU, Teaching Games for Understanding; SRQ-PE, Self-Regulation Questionnaire for Physical Education; AMS-PE, Academic Motivation Scale for Physical Education; BPNS-PE, Basic Psychological Needs for Physical Education; PSPP, Physical Self-Perception Profile; 2 × 2-AGF, 2 × 2 Achievement Goal Framework; SGS-PE, Social Goal Scale for Physical Education; PLOCS, Perceived Locus of Causality Scale; SDT, Self-Determination Theory; DFS-2, Dispositional Flow State; SMS, Sport Motivation Scale; PETE, Physical Education Teacher Education; LCQ, Learning Climate Questionnaire; EFI, Exercise Induced Feelings Inventory; BPNMS, Basic Psychological Needs Measurement Scale; MDCEQ, Motivation in Dance and Corporal Expression Questionnaire; PPECCS, Perceived Physical Education Class Climate Scale; ASCQ, Autonomy-Supportive Coaching Strategies Questionnaire; BPNES, Basic Psychological Needs in Exercise Scale; CL, Cooperative Learning; PLTA, Post-Lesson Teacher Analysis; TEGQ, Teacher's Emphasis on Goals Questionnaire; GCA, Games-Centered Approach; TGM, Tactical Games Model; SOFIT, System for Observing Fitness Instruction Time; PAIS, Physical Activity Intention Scale; LTEQ, Leisure-Time Exercise Questionnaire; MPPEQ, Metacognitive Process in Physical Education Questionnaire; LSSCL, Lesson Satisfaction Scale at the Contextual Level; CLA, Constraints-Led Approach; QEMPE, Questionnaire for Evaluating Motivation in Physical Education; PNTS, Psychological Need Thwarting Scale; SSI, Sport Satisfaction Instrument; IPAS, Intention to be Physically Active Scale; EBSS, Enjoyment/Boredom in Sport Scale; WVUTES, West Virginia Teaching Evaluation System; FS, Feeling Scale; S-PACES, Simplified Physical Activity Enjoyment Scale; AFSS, Activity Feelings State Scale*.

The asterisk (^*^) in certain references at the “Author(s) and year” column throughout Table 1 indicates that the study has also been quantitatively analyzed during the meta-analysis process, exposed in the following section *Meta-analysis findings*.

### Meta-Analysis Findings

Regarding the results of the meta-analysis, it is observed a significant overall results in fixed-effect model (*d* weighted effect size = 0.865; standard error = 0.062; 95% CI = 0.745, 0.986; *p* < 0.001) and random-effect model (*d* weighted effect size = 1.812; standard error = 0.584; 95% CI = 0.664, 2.956; *p* = 0.002). Hence, the standard difference means, and the CI of each study are showed in Table 2. However, the meta-analysis also showed significant heterogeneity in the *I*^2^ statistic (*I*^2^ = 98.834, *p* < 0.001).

**Table 2:** SDI meta-analysis about the comparison of DI approach and innovative MsBP in sport literacy at PE or formal educational context.

**References**	***d* effect size**	**SE**	**CI**		***p*-value**	**Forest plot**
			**Lower**	**Upper**		
Prusak et al. (2004)	0.104	0.309	−0.502	0.709	<0.737	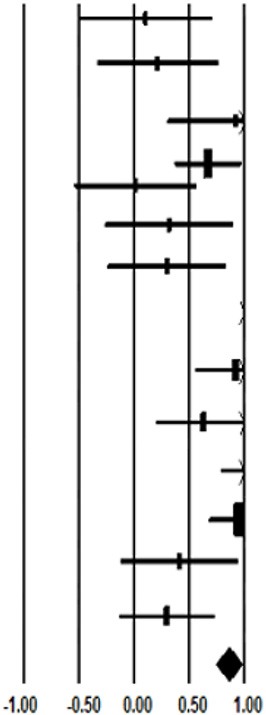
Wallhead and Ntoumanis (2004)	0.217	0.281	−0.334	0.767	0.441	
González-Cutre et al. (2011)	0.921	0.313	0.309	1.534	0.003	
Perlman (2011)	0.674	0.152	0.375	0.973	<0.001	
Perlman (2012b)	0.017	0.283	−0.537	0.572	0.952	
Amado et al. (2014)	0.321	0.297	−0.61	0.902	0.280	
Báguena-Mainar et al. (2014)	0.301	0.274	−0.236	0.838	0.272	
Chatzipanteli et al. (2015)	18.945	0.552	17.862	20.027	<0.001	
Chang et al. (2016)	0.927	0.188	0.560	1.295	<0.001	
Cuevas et al. (2016)	0.632	0.221	0.199	1.065	0.004	
Burgueño et al. (2017)	1.459	0.339	0.794	2.124	<0.001	
Fernández-Río et al. (2017)	0.949	0.134	0.685	1.212	<0.001	
Gil-Arias et al. (2017)	0.414	0.273	−0.120	0.948	0.129	
Chiva-Bartoll et al. (2018)	0.298	0.219	−0.132	0.728	0.174	
Overall	0.865	0.062	0.745	0.986	<0.001	

*SE, Standard error; CI, Confidence interval*.

In relation to the kind of models and pedagogical strategies implemented with the intention of comparing the impact of the motivational variables in sport content at PE or at training context (i.e., PETE), there are nine studies which applied one of the most MsBP widely used in PE classes, and five studies which implemented supportive-climate strategies in traditional sport lesson plans.

In this respect, the study which compared the CL with the DI, obtained a *d* weighted effect size = 0.949 (standard error = 0.134; *p* < 0.001). The two studies which compared the Hybridization of several MsBP (i.e., TGfU/SEM and CL/TGfU), showed a *d* weighted effect size = 0.344 (standard error = 0.171; *p* = 0.044). The five studies which analyzed the impact between the SEM and the DI, reported a *d* weighted effect size = 0.591 (standard error = 0.101; *p* < 0.001). The study which compared the TGfU and the DI obtained a *d* weighted effect size = 0.301 (standard error = 0.274; *p* = 0.272). The studies that analyzed the impact of the traditional sport content lessons using different supportive-climate contexts obtained a *d* weighted effect size = 1.740 (standard error = 0.137; *p* < 0.001). Finally, the study which analyse the TARGET strategy with the DI, showed a *d* weighted effect size = 0.921 (standard error = 0.313; *p* < 0.003).

## Discussion

The main purpose of this study was to summarize the research that had analyzed the influence of the MsBP and supportive-climate tenets on the students' motivation outcomes, including the degree of self-determined motivation, the satisfaction of the BPNs and the orientation through ego and task activities during PE sport literacy programs. Additionally, the second aim of the present investigation was to quantitative analyse the students' SDI results among quantitative and quasi-experimental studies that had compared some of the MsBP with the traditional DI approach, during sport PE lesson plans.

The concerns about the impact of the PE on the students' health and the acquisition of lifelong active habits had been studied since the theories of motivation were applied to the educational (Deci et al., 1991) and sport (Duda, 1992) contexts. Indeed, the first study that analyzed the factors that might influence the American and British students' intrinsic and extrinsic motivation was carried out by Biddle and Brooke (1992). Later, Goudas et al. (1994) carried out the first observational study in PE about the students' motivational orientations. In this study, he corroborated that motivation may be influenced by the nature of the specific program or the kind of sport. One year later, Goudas et al. (1995) observed that those student-centered teaching styles could significantly influence the goal involvement during a PE introduction to track and field lessons.

However, until the research of Clarke and Quill (2003), there were no studies that analyzed the impact of the MsBP on the students' perceptions, behaviors and motivations. They observed that SEM is an ideal framework to increase the students' involvement in PE lessons, in addition to students' understanding and performance at sport (specifically at athletics, soccer, netball, and sport acrobatics). One year later, Browne et al. (2004) carried out a research that compared the SEM with the DI approach, empathizing that an increase of responsibility and significant skills improvements were achieved in the SEM context.

The increase of literature about the comparison between innovative models and the traditional ones considerably increased in the last two decades. For this reason, this section discusses the results in several subsections facilitating the synthesis of the ideas to the reader.

### Implementation of Cooperative Learning and Its Impact on the Students' Motivation

CL in PE (Barrett, 2005) is a model that optimizes the learning outcomes according to five important elements (Johnson and Johnson, 1994): (I) positive interdependence, (II) positive face-to-face interaction, (III) group processing, (IV) interpersonal and small-group skills, and (V) individual accountability. On the other hand, Pujolás (2008) and Dyson et al. (2010) emphasized that time is also a very important factor to bear in mind when CL is implemented because it is observed that the degree of cooperation in a team is directly related to the quantity of time dedicating in working together. Regarding the psychosocial outcomes using this model, two studies (i.e., Goodyear et al., 2014; Fernández-Río et al., 2017) have investigated qualitative and quantitatively the effects of the CL among secondary PE students.

After implementing a CL unit using flip cameras, Goodyear et al. (2014) observed positive learning environments where the students' responsibility, collaboration, and cooperation were reinforced. As it is also observed in the adult population (Wang, 2012), CL creates more successful experiences that increase the self-determined motivation. In addition, Goodyear et al. (2014) reinforced the idea of implementing this model using roles (as in SEM) because non-sporty participants can be more engaged in PE.

Most recently, Fernández-Río et al. (2017) compared the impact of different life-style activities and sports units using CL and DI in a mixed study (i.e., both quantitative and qualitative). In this research, it was observed a significant cooperative class climate among the students who participated in the CL group. Otherwise, novelty was a positive variable that influenced the students' self-determined motivation. However, students also reported certain disappointment when sometimes several students did not work cooperatively in the CL group. Indeed, in spite of the fact that CL could be difficult to apply in certain contexts, teachers should be aware of the benefits that produce (Goodyear and Casey, 2015) in conceptual, attitudinal and procedural content. For those reasons, it is confirmed the idea that pedagogical and social factors have an impact on psychological mediators that determined the different types of motivation.

### Implementation of Constraint-Led Approach and Its Impact on the Students' Motivation

CLA is also situated in the non-linear pedagogy framework (Davids et al., 2005). This model is based on the ecological dynamics theory. It establishes that movement patterns are organized under the interaction of constraints (Renshaw and Chow, 2019). In this sense, this model emphasizes the necessity of creating environments to promote movement patterns according to the unique individual physical and psychological characteristics or profiles. According to Chow et al. (2011), CLA is very similar to the application of the Modify Games or Small-Sided (and Conditioned) Games at TGfU [encompassed in the GCA]. However, the main difference between CLA and TGfU is that this approach is theoretically developed in the ecological dynamics of the non-linear pedagogy (Renshaw et al., 2015).

Although, there is no research that analyzed the benefits of the CLA on the students' motivation or the BPNs satisfaction in the educational context (Tan et al., 2012; Moy et al., 2016) analyzed this approach comparing the psychological effects with the traditional DI approach in pre-service PE teachers. They reported that the use of CLA increased the pre-service teacher students' tactical/technical intelligence, as well as the intrinsic motivation. In this sense, it was confirmed that perceived competence is positively associated with the intrinsic motivation. Additionally, the study concluded that the implementation of non-linear pedagogy through CLA alongside effective verbal instruction and positive feedback promote not only the acquisition of determinant skills, but also it can produce an increase of personal effort, enjoyment, interest, and excitement among students. Indeed these outcomes might determine a positive effect on students' task engagement and persistence for practice both in educational and extracurricular context.

### Implementation of Games-Centered Approach and Its Impact on the Students' Motivation

CGA is a “great” framework that reinforces the game understanding and the technical skills (i.e., tactical/technical intelligence) via implementing Modify Games or Small-Sided (and Conditioned) Games adapted to the characteristics of the students (Harvey and Jarrett, 2014). That is to say, technical abilities (prioritized in the DI approach) are developed when a tactical problem arises in the game (Werner et al., 1996). In this way, those models which provide and facilitate the sport content understanding through games are encompassed in this approach. Hence, in the present study, four different types of models (i.e., TIG, TGfU, TASG, and TGM) encompassed in this approach were identified.

Gray et al. (2009) showed that the implementation of the TIG, in contrast to the traditional approaches, increases the opportunities to play the game, and consequently, improves the students' decision-making intelligence. Similarly, Smith et al. (2014) observed an increase in the amount of moderate-to-vigorous physical activity among TGM students in contrast to DI students. However, when these results are divided by gender, discrepancies are observed: female TGM students do not meet the 50% of physical activity level recommended for PE sessions (Hartwig et al., 2019).

On the other hand, Jones et al. (2010) highlighted that using TGfU, in contrast to the traditional skill-based approach, also produces an increase of fun and enjoyment due to the fact that students perceive more autonomous environments. Similar results had been highlighted by Mandigo et al. (2008), who reported an increase in students' intrinsic motivation using the TASG. Indeed, Báguena-Mainar et al. (2014) emphasized that participants perceived more responsibility when the pedagogical frameworks based on the student-centered approach were implemented, producing greater levels of autonomy and satisfaction. Recently, Harvey et al. (2017) also observed that the implementation of contextualized games situations determined the enjoyment and motivation of the students. On the contrary, Smith et al. (2014) did not found significant differences in the students' intrinsic motivation when they compared several sports using the TGM, suggesting that the teacher behaviors and the time of instruction might influence these results.

In this respect, Mandigo et al. (2008) proposed to reinforce the autonomy-supportive climate when TGfU is going to be implemented in PE classes. In addition, their findings supported the idea that the intrinsic motivational levels among girls could significantly increase when they experience autonomy-supportive environments. On the other hand, taking into account the teacher behavior and the teaching style, Gray et al. (2009) suggested implementing the Epstein's (1989) TARGET structure alongside the MsBP. Finally, Harvey et al. (2017) corroborated the idea that providing choices during the implementation of the model reduced the teachers' controlling behaviors.

Finally, every study (Mandigo et al., 2008; Gray et al., 2009; Jones et al., 2010; Báguena-Mainar et al., 2014; Harvey et al., 2017) coincides in the idea that CGA is beneficial to increase the most self-determined form of motivation taking into account the autonomy support climate. However, as Harvey et al. (2017) indicated, it is necessary to increase the commitment to this approach to reduce the controlling teacher behaviors that influence negatively on the students' motivation.

### Implementation of Sport Education Model and Its Impact on the Students' Motivation

The SEM is a pedagogical framework with seven features (i.e., seasons, formal competition, affiliation to a unique team, data recording, festivity, application of roles, and final competition) aims to produce an authentic sport experience simulating the real aspects of the game, but adapting every element related to the sport itself to the educational context (Siedentop et al., 2019).

SEM has been the most widely used model for analyzing the impact on the students' psychological variables and their effects on the students' sport adherence and lifelong active habits. Clarke and Quill (2003) observed positive perceptions among students after experience a SEM season. In this sense, they identified that the sense of belonging to a team, as well as the increase of the responsibility, produced an increase in the motivation to practice games. Similarly, Browne et al. (2004); Perlman (2010), as well as Wallhead and Ntoumanis (2004), highlighted that the affiliation (to a unique team) is an important feature to deliver supportive and mastery-climate. In the same line, O'Donovan (2003) also confirmed that the implementation of SEM increases the students' motivation and task-climate.

On the other hand, Wallhead and Ntoumanis (2004) found that one feature of the SEM was that formal competition can negatively influence the students' self-determined motivation and the ego-involving climate. They proposed several teaching strategies to counteract those negative effects (e.g., seasons related to tasks such as choreographies or fair-play assessment). However, as Hastie and Sinelnikov (2006) explained, the aforementioned feature can be considered as a key element of how to improve skills. They reported the fact that training to improve the skills of the team in order to win games produced enjoyment. In their study, they also observed that other features such as the roles and the affiliation to a team also produced an increase of enjoyment and intrinsic motivation.

When SEM is compared with traditional approaches, some authors (Spittle and Byrne, 2009; Perlman, 2010, 2011) have reported an increase of enjoyment and the BPNs satisfaction when students experienced the SEM. Specifically, Perlman (2010) spotlighted that amotivated PE students increased their engagement and enjoyment to PE classes during the SEM season. In this regard, these students reported an increase in relatedness, fostered by the features of the model itself. In the same year, Perlman and Goc-Karp (2010) qualitatively reported that the three psychological needs can be also satisfied using the SEM. However, in a posterior study, Perlman (2011) did not observe significant changes in the perception of autonomy and competence, possibly because of the prescription of learning experiences implemented in that season.

The SEM can also be implemented with the TARGET structure (Epstein, 1989), as Hastie et al. (2014) demonstrated. In this case, it is demonstrated that the teacher has to manipulate the SEM to orientate the needs of each student to create a mastery-oriented climate, and consequently, to produce more self-determined forms of motivation. In this sense, Medina-Casaubón and Burgueño (2017) also confirmed that SEM helps students to develop their holistic emotional, psychological and social intelligence, together with the acquisition of the sport competence (i.e., tactical/technical skills).

Recently, Cuevas et al. (2016) confirmed that the intrinsic motivation was significantly higher in the students who experienced the SEM in contrast with the students who participated in the DI, verifying the idea that SEM can produce enjoyment, pleasure, and well-being. These factors can determine the way in which the effort variable increases. This was the first study that analyzed empirically the thwarting of the BPNs, that is to say, the negative effect due to a hostile context (Bartholomew et al., 2011). However, they observed a slight decrease in thwarting competence among SEM students. On the other hand, it is also be confirmed that SEM produces high levels of self-determined motivation that directly and positively impacts on the adherence to continue practicing a sport or a healthy activity.

### Implementation of Hybridizations and Its Impact on the Students' Motivation

Hybridizations of MsBP might be the solution to extend the benefits of implementing single MsBP (González-Víllora et al., 2018). However, it is also supported the idea of combining single MsBP or parts of them.

In the present study, there were identified two comparisons between the hybridization of two models with the traditional DI approach. Thus, Gil-Arias et al. (2017) investigated the impact of the hybridization of the TGM/SEM on the most self-determined motivation as well as the satisfaction of the BPNs. Their methodology was a cross over or counterbalance design (i.e., one group participated in the hybridization unit whereas the other group participated in the DI unit, later the first group experienced the DI unit and the second one the hybridization unit), which demonstrated that using the hybrid TGfU/SEM increased the students tactical/technical intelligence. In addition, the authors found that the sense of belonging or unit (a feature of the SEM; affiliation) was higher in the first group (which experienced the hybridization first). Regarding the motivational variables, it could not be confirmed that students from group one significantly improved their self-determined motivation. In this sense, it was also observed that group one obtained lower BPNs when they experienced DI after the hybridization.

Otherwise, the recent study of Chiva-Bartoll et al. (2018) also confirmed that hybridizations (in this case TGfU/CL hybridization) can impact on the task-involving climate. In addition, this study also confirmed the idea of Smith et al. (2014) who proposed that the teacher behavior and pedagogical strategies could provide a mastery-oriented climate, because statistical differences were not found in the progression of the motivational climate between hybridization and traditional approaches. In this sense, Chiva-Bartoll et al. (2018) reinforced the idea of applying reciprocal and guided discovery teaching styles to optimize the student self-determined motivation, autonomy and mastery-climate alongside innovative approaches.

Both studies (Gil-Arias et al., 2017; Chiva-Bartoll et al., 2018) suggested that hybridizations have a positive impact on the self-determined motivation and the BPNs satisfaction, in contrast to traditional approaches that prioritizes the decontextualized technical skills learning.

### Implementation of Autonomy-Supportive Climate and Its Impact on the Students' Motivation

Although MsBP are an ideal context to obtain more self-determined forms of motivation, it is observed that the teacher behavior and climate could definitely impact on the students' psychological outcomes (Gray et al., 2009; Hastie et al., 2014). In this sense, there are eight researches (Prusak et al., 2004; Lonsdale et al., 2009; González-Cutre et al., 2011; Gillison et al., 2013; Amado et al., 2014; Chatzipanteli et al., 2015; Chang et al., 2016; and Vazou et al., 2019) that analyses the impact of the support-climate on the self-determined motivation, enjoyment and BPNs satisfaction during a traditional sport and/or life-style activities units.

The first idea that Prusak et al. (2004); Lonsdale et al. (2009) and Chang et al. (2016) observed was that students are more self-determined motivated when options to choice (e.g., activity, duration, or classmate) are given to them. In this sense, Lonsdale et al. (2009) highlighted that in this kind of autonomy-supportive climates, the self-determined motivation increases in contrast to teacher-centered approaches. However, as Gillison et al. (2013) indicated, some kinds of choice with null structure may undermine the positive forms of motivations. Otherwise, implementing reciprocal and inclusion teaching styles in traditional sessions can produce a significant increase of declarative and procedural knowledge whereas the students' intrinsic motivation also increases (Chatzipanteli et al., 2015). Finally, Chang et al. (2016) demonstrated that autonomy-supportive sessions can be adapted to the circumstances of the context to optimize the students' self-determined motivation.

On the other hand, González-Cutre et al. (2011) highlighted that the TARGET structure in PE units enables to increase the task-involving climates, and consequently, the desire to continue practicing physical and sport activities with an increase of motivation in PE. Recently, Vazou et al. (2019) emphasized the importance of providing contexts where the students perceive enjoyment and competence that engage them to be continuously involved in physical activities. With this purpose, they proposed using a wide range of resources (e.g., music or videos), as well as an increase of the student-centered pedagogy programs even when the lesson plans are related to a fitness program or any other health-life activity.

## Practical Application

It is important to analyse the content and the pedagogical strategies which pursuit an optimal and holistic children's affective, cognitive, and physical development to be applied in PE classes. In this sense, it was recently observed that MsBP, and specifically, GCA maximize the acquisition of the motor and sport competence among PE programs during sport literacy contents (González-Víllora et al., 2019). In this study, it was demonstrated that MsBP and autonomy-supportive classes also foster the self-determined motivation in children. This fact impacts directly on the engagement and adherence to maintain active lifestyle habits, e.g., go walking or joining to a futsal club because the student perceived a positive enjoyment when the futsal PE unit was implemented (Morgan et al., 2005).

**Figure 3:**
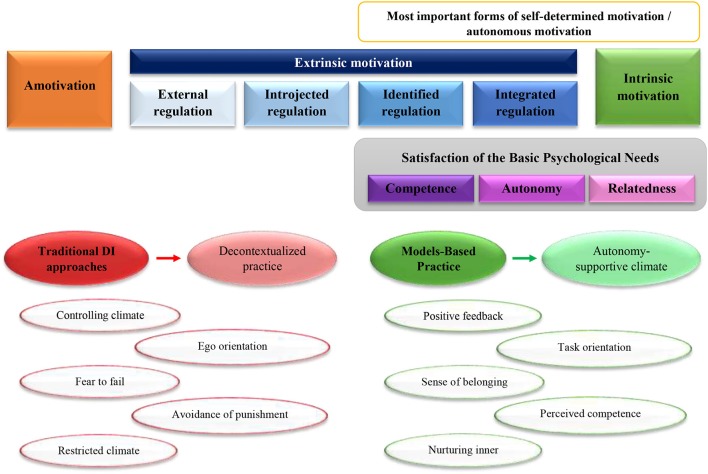
How to promote motivation in PE context? Applying the SDT and the BPNs frameworks.

As it is showed in Figure 3, the motivation continuum is not a stable characteristic of the human behavior (Ryan and Deci, 2017). It is influenced by external factors and can be changed positively or negatively over time.

In the continuum the most self-determined or autonomous types of motivation are (I) identified regulation, (II) integrated regulation and finally, (III) intrinsic motivation (please, see subsection *The Self-Determination Theory (SDT) and Basic Psychological Needs (BPNs)* to read one example of these kinds of regulation in an educational context). In this systematic review, it is observed how this kind of motivation can be supported by the implementation of MsBP. However, as Hastie et al. (2014) it is important to incorporate an autonomous supportive environment which surrounded the application of the MsBP. Indeed, as Gillison et al. (2013) highlighted, it is very important to satisfy the BPNs of the students giving informational feedback using a positive intonation, showing empathy and engage students to be involved in a game or activity, or provided a credible rationale of why the targeted activity or game is important (i.e., identified regulation).

On the other hand, every comparative study analyzed in this work highlighted that traditional DI approaches impact negatively on the most self-determined forms of motivation, that is to say, influence positively on the less self-determined forms of motivation (i.e., amotivation, external regulation and introjected regulation). Indeed, Ntoumanis et al. (2004) and Huhtiniemi et al. (2019) observed that students who do not perceive enjoyment in the PE classes (normally in traditional classes) are more likely to be amotivated. This fact would be worsened if students perceived less competence when they are involved in skill-based drills. For that reason, Mandigo et al. (2019) has recently observed that physical literacy and sport competence can be increased if both primary and secondary education students are engaged and exposed to multiple forms of physical and sport activities through MsBP such as TGfU in contrast to traditional sport specialization or stimulation of isolated and repeated games.

In summary, teachers (or coaches) should select the best pedagogical strategy according to the main features of the students, content, curriculum and contest. In this sense, PE teachers should focus on developing comprehensive students' physical literacy and *sport competence* through MsBP, but also they should be aware about the positive influence of this kind of strategies on the psychosocial variables that directly impact on the students' self-determined motivation, and consequently on the adherence of active lifestyle.

## Conclusions

This secondary research examines the impact of MsBP programs and autonomous supportive climates in PE on the student's psychosocial outcomes, including the level of motivation. Although more scientific literature is needed in this field, it is clearly observed that students' self-determined motivation increased when MsBP are implemented or when traditional DI sessions are carried out using a plethora of autonomous supportive pedagogical resources. What is more, MsBP are ideal pedagogical frameworks to produce significant increases of (I) the *sport competence* and (II) the self-determined motivation among PE students in contrast to traditional DI environments.

On the contrary, it is also observed that MsBP are not intrinsically pedagogical strategies to engage the practice of physical activities or sports beyond the PE classes. In this sense, models need (I) to be adapted to the characteristics and necessities of each context (including students, materials, contents, curricular elements, specific contexts, and teachers), and (II) to incorporate autonomous supportive pedagogical strategies to promote students self-determined motivation alongside the development of an optimal level of motor and sport competence, which enables students to have an active lifelong habits.

## Data Availability Statement

All datasets generated for this study are included in the manuscript/supplementary files.

## Author Contributions

MS-D and SG-V were involved in the conception and design of the systematic review and executed the meta-analysis. Firstly, MS-D elaborated the protocol registering in PROSPERO (https://www.crd.york.ac.uk/prospero/), once the rest of the authors (SG-V, JP-V, and GL-S) had reviewed and improved it. Finally, the four authors carried out the systematic search and write the article.

### Conflict of Interest

The authors declare that the research was conducted in the absence of any commercial or financial relationships that could be construed as a potential conflict of interest.
